# Prognostic value of sarcopenia in patients with nasopharyngeal carcinoma: a meta-analysis

**DOI:** 10.1080/07853890.2025.2530695

**Published:** 2025-07-11

**Authors:** Xiaobin Gu, Yonggang Shi

**Affiliations:** Department of Radiation Oncology, The First Affiliated Hospital of Zhengzhou University, Zhengzhou, China

**Keywords:** Sarcopenia, nasopharyngeal carcinoma, biomarker, evidence-based medicine, meta-analysis

## Abstract

**Background:**

Sarcopenia is widely explored for its significance for predicting nasopharyngeal carcinoma (NPC) prognosis, yet current results are inconsistent. Consequently, this meta-analysis focused on investigating its precise role in predicting NPC prognosis.

**Methods:**

We systemically retrieved studies in PubMed, Web of Science, Embase and Cochrane Library from inception to 17 March 2025. Sarcopenia was analysed for its role in predicting overall survival (OS) and progression-free survival (PFS) in NPC through determining combined hazard ratios (HRs) and 95% confidence intervals (CIs). Thereafter, associations of sarcopenia with NPC clinicopathological factors were estimated through aggregating odds ratios (ORs) with 95%CIs.

**Results:**

There were eight studies with 5458 cases enrolled in this study. Based on the aggregated findings, sarcopenia was remarkably related to unfavourable OS (HR = 1.88, 95% CI = 1.47–2.40, *p* < .001) as well as PFS (HR = 2.03, 95%CI = 1.48–2.80, *p* < .001) of NPC. Moreover, sarcopenia was significantly associated with higher T stage (OR = 1.33, 95%CI = 1.12–1.59, *p* = .002). But it was not related to gender (OR = 0.87, 95%CI = 0.42–1.77, *p* = .694), N stage (OR = 1.00, 95%CI = 0.87–1.16, *p* = .952) and tumour-node-metastasis (TNM) stage (OR = 1.17, 95%CI = 0.94–1.46, *p* = .153) in NPC.

**Conclusions:**

This meta-analysis demonstrated that sarcopenia was a significant prognostic marker for poor OS and PFS in patients with NPC. Moreover, sarcopenia was also significantly associated with higher T stage of NPC.

## KEY MESSAGES

To our knowledge, the current work provides the first meta-analysis investigating the value of sarcopenia for predicting NPC prognosis.

Sarcopenia was a significant prognostic marker for poor OS and PFS in patients with NPC.

Sarcopenia was also significantly associated with higher T stage of NPC.

## Introduction

Nasopharyngeal carcinoma (NPC) is typically connected to the Epstein–Barr virus and has a distinct geographic distribution, with the highest occurrence in certain Asian regions [1]. According to GLOBOCAN, there were 120,416 new NPC cases and 73,476 death cases due to this disease in 2022 globally [2]. Induction chemotherapy plus concurrent chemoradiotherapy (CCRT) serves as the typical method to treat locally advanced NPC [3]. However, bone, liver and lung metastatic lesions are detected in approximately 6–15% of NPC cases when they are first diagnosed [4]. Although there have been improvements in diagnostic techniques and treatment strategies, managing advanced NPC continues to be difficult, indicating a need for additional research to better patient outcomes. The median overall survival (OS) is under 2 years for around 15–30% of metastatic or recurrent NPC cases [5]. Thus, detecting new and reliable biomarkers related to NPC prognosis is imperative.

Sarcopenia, as first defined in 1989, is a syndrome characterized by a widespread and progressive reduction in function and mass of skeletal muscles. In line with the European Working Group on Sarcopenia in the Elderly in 2010, sarcopenia is identified by low muscle mass, diminished physical fitness and decreased muscle strength [6]. Determining a reduction in skeletal muscle mass is necessary for diagnosing sarcopenia, and several tools and techniques are used for this objective [7]. Computed tomography is utilized in both research and clinical settings to assess the mass of skeletal muscle and fat tissues in the body [8]. The measurement of skeletal muscle mass at the third lumbar vertebra (L3) shows high correlation with the total skeletal muscle mass, establishing the CT-defined skeletal muscle index (SMI) as the diagnostic gold standard for sarcopenia [9].

Sarcopenia is extensively suggested with a significant value in predicting tumour prognosis, such as non-small cell lung [10], oesophageal [11], breast cancers [12], hepatocellular carcinoma [13] and gastric cancer [14]. Previous studies have analysed sarcopenia for its effect on predicting NPC prognosis, but findings remained controversial [15–22]. For instance, in certain studies, sarcopenia was remarkably related to poor prognosis of NPC [17–20]. However, according to other researchers, sarcopenia was not markedly related to survival outcomes of NPC [15,16,21]. Thus, the current meta-analysis identified the value of sarcopenia for estimating NPC prognostic outcome, and analysed the connection between sarcopenia and NPC clinicopathological features.

## Materials and methods

### Study guideline

This work conformed to the Preferred Reporting Items for Systematic Reviews and Meta-Analyses (PRISMA) guideline [23].

### Literature search

A thorough search of PubMed, Embase, Web of Science, and Cochrane Library was conducted till 17 March 2025 using the search strategies below: (sarcopenia OR sarcopenias OR sarcopenic) AND (nasopharyngeal carcinoma OR NPC OR nasopharyngeal cancer). Both Medical Subject Headings (MeSH) terms and free works were utilized. Just English articles were considered. References from these articles were carefully examined for additional reports.

### Inclusion and exclusion criteria

Studies below were included: (1) NPC was confirmed based on pathology; (2) articles investigated the correlation of sarcopenia with survival of NPC; (3) sarcopenia was diagnosed by SMI or other standards; (4) hazard ratios (HRs) with 95% confidence intervals (CIs) were available; (5) the threshold was identified to stratify sarcopenia and non-sarcopenia; (6) cohort studies, including prospective and retrospective cohorts; (7) NPC patients received any treatments according to the treatment guidelines; (8) English articles. Studies below were excluded: (1) case reports, meeting abstracts, reviews, comments and letters; (2) those with duplicate cases; and (3) animal studies.

### Data extraction and quality assessment

Two researchers (X.G. and Y.S.) separately obtained information in eligible studies, and disagreements were settled by discussion. Data below were isolated: author, year, country, sample size, age, gender, study design, study duration, study centre, tumour-node-metastasis (TNM) stage, treatment, sarcopenia criteria, threshold, threshold determination approach, prevalence of sarcopenia, survival analysis, survival outcomes, follow-up, HRs and 95%CIs. With univariate and multivariate regression being carried out, HRs were first extracted from the multivariate analysis. OS and progression-free survival (PFS) were the primary and secondary endpoints, separately. Literature quality was analysed by using Newcastle–Ottawa Scale (NOS), which focuses on three primary areas: patient selection, outcome assessment and comparability [24]. NOS scores were 0–9, with ≥6 suggesting high-quality.

### Statistical analysis

We analysed sarcopenia’s effect on predicting OS and PFS of NPC through computing combined HRs and 95%CIs, and employed Cochran’s *Q* statistic and the *I*^2^ statistics to evaluate among-study heterogeneities. Significant heterogeneity was indicated by an *I*^2^ >50% along with a *p* value less than .10. In these situations, the study results were aggregated using the random-effects model; or else, the fixed-effects model should be utilized. We carried out subgroup analysis to detect the source of heterogeneity. Odds ratios (ORs) with 95%CI were aggregated for estimating associations of sarcopenia with NPC clinicopathological characteristics. We carried out sensitivity analysis for determining whether our combined findings were stable through removing each article one at a time to observe its effect on the overall estimate. Publication bias was evaluated by using Begg’s test and Egger’s test. Stata software version 12.0 (Stata Corp., College Station, TX) was employed for statistical analysis. *p* < .05 stood for significant difference.

## Results

### Literature search procedure

**Figure 1.**
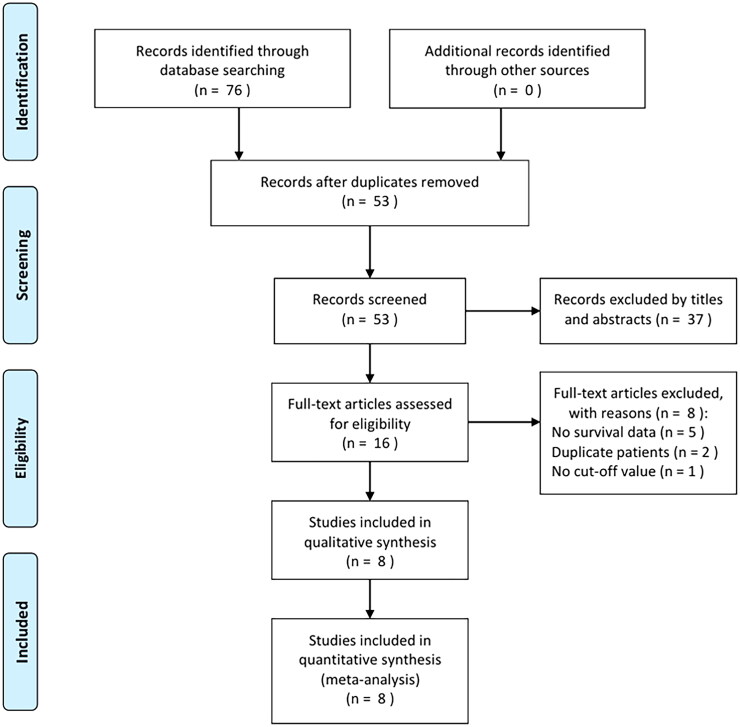
PRISMA flow diagram of study selection.

Through primary search, 76 articles were acquired, and 53 were retained after duplicates were eliminated (Figure 1). Upon title- and abstract-reading, we eliminated 37 articles due to animal studies and irrelevance. Subsequently, full-texts of 16 articles were read, including eight being excluded due to unavailable survival information (*n* = 5), duplicate patients recruited (*n* = 2) and unavailable threshold identified (*n* = 1). Finally, eight studies with 5458 patients [15–22] were recruited into this work (Figure 1).

### Enrolled study features

All enrolled articles [15–22] were published from 2019 to 2025 and were all published in English (Table 1). There were six articles carried out in China [15–18,20,22], whereas one each in Turkey [19] and Japan [21], respectively. The sample sizes were 64–1767 (median, 511.5). All included studies were of retrospective design. There were seven single centre studies [15–17,19–22], whereas one multicentre trial [18]. All included studies treated patients with CCRT. Seven studies used SMI to diagnose sarcopenia [15–18,20–22] and one study applied total masseter muscle volume (TMMV) [19]. The thresholds of males and females are presented in Table 1. Five studies determined the cut-off values according to literature [15,16,18,20,21] and three studies used receiver operating characteristic (ROC) curve [17,19,22]. The prevalence of sarcopenia in NPC patients ranged from 24.4% to 43.3% in included studies. All eight articles mentioned the role of sarcopenia in predicting OS [15–22], whereas three reported the relation of sarcopenia with PFS [18–20] in NPC. Five studies derived HRs and 95%CIs upon multivariate regression [17–20,22] and three studies used univariate analysis [15,16,21]. The NOS scores were 7–9, suggesting their high quality [15–22] (Table 1; Supplemental file 1).

**Table 1.** Basic characteristics of included studies in this meta-analysis.

Study	Country	Sample size	Gender (M/F)	Age (years) Median (range)	Study period	Study centre	TNM stage	Treatment	Sarcopenia criteria	Cut-off value		Cut-off determination	Prevalence of sarcopenia	Survival outcome	Follow-up (months) Median	Survival analysis	NOS score
										For male	For female						
Huang et al. [15]	China	394	298/96	46 (18–79)	2015–2017	Single centre	I–IVb	CCRT	SMI	40.8 cm^2^/m^2^	34.9 cm^2^/m^2^	Literature	33.0%	OS	22.7 (2.5–46.4)	Univariate	8
He et al. [16]	China	1767	1382/385	<60 y: 1539 ≥60 y: 228	2008–2013	Single centre	I–IVb	CCRT	SMI	52 cm^2^/m^2^	38 cm^2^/m^2^	Literature	38.7%	OS	77.6	Univariate	9
Hua et al. [17]	China	806	602/204	45 (18–84)	2010–2014	Single centre	II–IV	CCRT	SMI	22 cm^2^/m^2^	18.6 cm^2^/m^2^	ROC curve	24.4%	OS	1–60	Multivariate	8
Liu et al. [18]	China	1307	917/390	48 (18–80)	2010–2019	Multicentre	II–IVa	CCRT	SMI	55 cm^2^/m^2^	39 cm^2^/m^2^	Literature	37.2%	OS, PFS	61	Multivariate	9
Pehlivan et al. [19]	Turkey	97	69/28	59 (19–79)	2010–2022	Single centre	II–IVa	CCRT	TMMV	38.0 cm^3^	38.0 cm^3^	ROC curve	43.3%	OS, PFS	63 (6–141)	Multivariate	7
Liu et al. [20]	China	545	396/149	46 (18–69)	2016–2019	Single centre	II–IVa	CCRT	SMI	51 cm^2^/m^2^	38 cm^2^/m^2^	Literature	42.8%	OS, PFS	54 (2–100)	Multivariate	8
Ichinose et al. [21]	Japan	64	55/9	57	2005–2022	Single centre	II–IVb	CCRT	SMI	45.2 cm^2^/m^2^	40.9 cm^2^/m^2^	Literature	40.6%	OS	1–120	Univariate	8
Yang et al. [22]	China	478	324/154	45 (18–76)	2016–2019	Single centre	III–IVa	CCRT	SMI	12.8 cm^2^/m^2^	11.2 cm^2^/m^2^	ROC curve	37.9%	OS	59 (9–92)	Multivariate	8

TNM: tumor-node-metastasis; SMI: skeletal muscle index; TMMV: total masseter muscle volume; ROC: receiver operating characteristic; OS: overall survival; PFS: progression-free survival; CCRT: concurrent chemoradiotherapy; M: male; F: female; NOS: Newcastle–Ottawa Scale.

### Sarcopenia and OS

**Figure 2.**
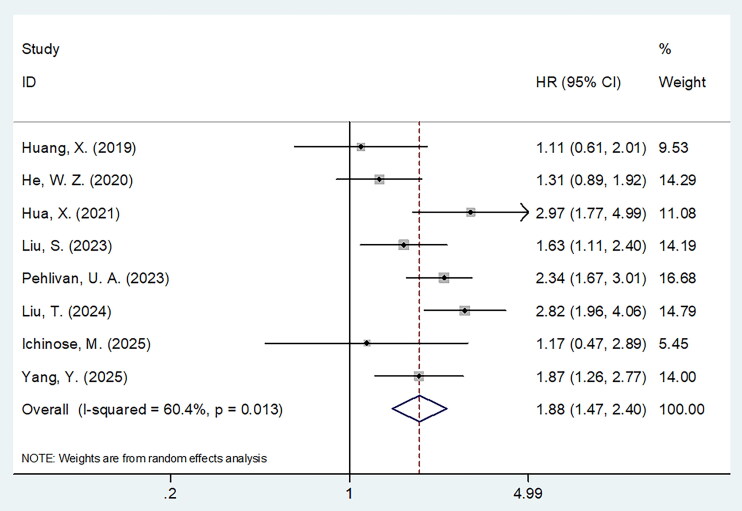
Forest plots for the prognostic role of sarcopenia for OS in patients with NPC. According to HR = 1.88, 95%CI = 1.47–2.40 and *p* < .001, sarcopenia was closely related to poor OS of NPC.

Eight articles with 5458 patients [15–22] reported sarcopenia for its role in predicting OS of NPC. Due to the obvious heterogeneity (*I*^2^ = 60.4%, *p* = .013), we applied the random-effects model. According to HR = 1.88, 95%CI = 1.47–2.40 and *p* < .001, sarcopenia was closely related to poor OS of NPC (Table 2; Figure 2). Based on subgroup analysis, sarcopenia exerted its significant effect on predicting OS independent from country, sample size, study centre, definition criteria or cut-off determination method (*p* < .05; Table 2).

**Table 2.** Subgroup analysis of prognostic value of sarcopenia for OS in patients with NPC.

Subgroups	No. of studies	No. of patients	Effects model	HR (95%CI)	*p*	Heterogeneity	
						*I*^2^ (%)	Ph
Total	8	5458	Random	1.88 (1.47–2.40)	<.001	60.4	0.013
Country							
China	6	5297	Random	1.85 (1.37–2.49)	<.001	66.1	0.011
Others	2	161	Random	1.90 (1.02–3.54)	.043	50.6	0.155
Sample size							
<500	4	1033	Random	1.74 (1.23–2.45)	.002	51.1	0.105
≥500	4	4425	Random	2.03 (1.36–3.02)	.001	73.9	0.009
Study centre							
Single centre	7	4151	Random	1.91 (1.45–2.53)	<.001	64.1	0.010
Multicentre	1	1307	–	1.63 (1.11–2.40)	.013	–	–
TNM stage							
I–IV	2	2161	Fixed	1.25 (0.90–1.72)	.181	0	0.651
II–IV	5	2819	Fixed	2.27 (1.89–2.72)	<.001	45.4	0.120
III–IV	1	478	–	1.87 (1.26–2.77)	.002	–	–
Sarcopenia criteria							
SMI	7	5361	Random	1.79 (1.35–2.38)	<.001	61.9	0.015
TMMV	1	97	–	2.34 (1.74–3.14)	<.001	–	–
Cut-off determination							
Literature	5	4077	Random	1.60 (1.11–2.30)	.013	66.4	0.018
ROC curve	3	1381	Fixed	2.28 (1.84–2.83)	<.001	0.8	0.365
Survival analysis							
Univariate	3	2225	Fixed	1.24 (0.91–1.68)	.169	0	0.896
Multivariate	5	3233	Fixed	2.24 (1.90–2.65)	<.001	35.3	0.186

TNM: tumor-node-metastasis; SMI: skeletal muscle index; TMMV: total masseter muscle volume; ROC: receiver operating characteristic; OS: overall survival; NPC: nasopharyngeal carcinoma.

### Sarcopenia and PFS

**Figure 3.**
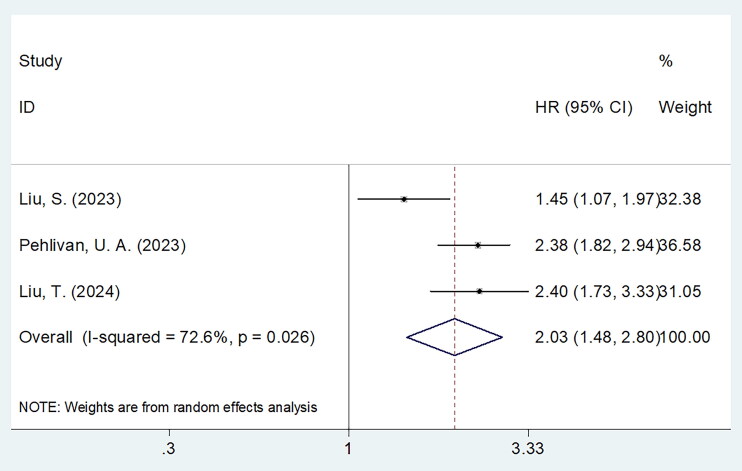
Forest plots for the prognostic role of sarcopenia for PFS in patients with NPC. From the aggregated results, sarcopenia significantly predict poor PFS of NPC (HR = 2.03, 95%CI = 1.48–2.80, *p* < .001).

Three articles comprising 1949 cases presented the correlation between sarcopenia and PFS [18–20] in NPC. Considering the obvious heterogeneity (*I*^2^ = 72.6%, *p* = .026), we utilized the random-effects model. From the aggregated results, sarcopenia significantly predict poor PFS of NPC (HR = 2.03, 95%CI = 1.48–2.80, *p* < .001; Table 3; Figure 3). From subgroup analysis, sarcopenia was still a prominent prognostic biomarker related to PFS despite country, study centre or definition criteria (all *p* < .05; Table 3).

**Table 3.** Subgroup analysis of prognostic value of sarcopenia for PFS in patients with NPC.

Subgroups	No. of studies	No. of patients	Effects model	HR (95%CI)	*p*	Heterogeneity	
						*I*^2^ (%)	Ph
Total	3	1949	Random	2.03 (1.48–2.80)	<.001	72.6	0.026
Country							
China	2	1852	Random	1.86 (1.13–3.05)	.014	79.5	0.027
Others	1	97	–	2.38 (1.87–3.02)	<.001	–	–
Study centre							
Single centre	2	642	Fixed	2.39 (1.97–2.90)	<.001	0	0.968
Multicentre	1	1307	–	1.45 (1.07–1.97)	.018	–	–
Sarcopenia criteria							
SMI	2	1852	Random	1.86 (1.13–3.05)	.014	79.5	0.027
TMMV	1	97	–	2.38 (1.87–3.02)	<.001	–	–

PFS: progression-free survival; NPC: nasopharyngeal carcinoma.

### The correlation between sarcopenia and clinicopathological factors

**Figure 4.**
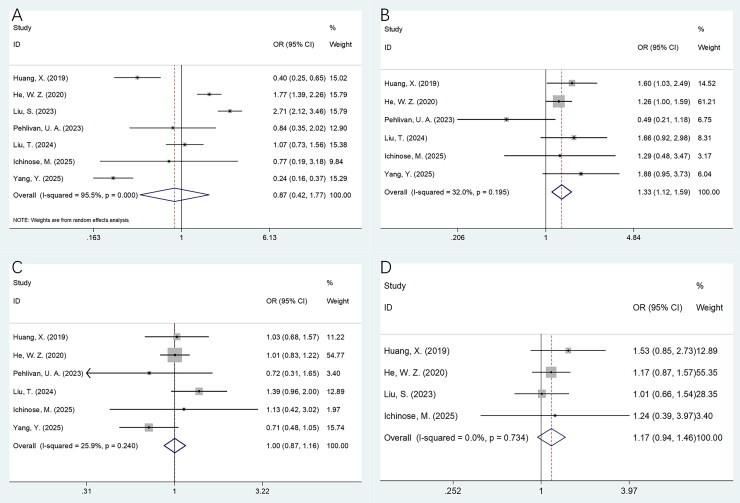
Forest plots presenting the association between sarcopenia and clinicopathological features of NPC. (A) Gender (male vs. female); (B) T stage (T3–T4 vs. T1–T2); (C) N stage (N2–N3 vs. N0–N1); and (D) TNM stage (III–IV vs. I–II).

Seven studies consisting of 4652 patients [15,16,18–22] provided the data on association between sarcopenia and clinicopathological factors of NPC. The pooled data showed that sarcopenia was significantly correlated to higher T stage (OR = 1.33, 95%CI = 1.12–1.59, *p* = .002; Table 4; Figure 4). But sarcopenia was unrelated to gender (OR = 0.87, 95%CI = 0.42–1.77, *p* = .694), N stage (OR = 1.00, 95%CI = 0.87–1.16, *p* = .952) and TNM stage (OR = 1.17, 95%CI = 0.94–1.46, *p* = .153) (Table 4; Figure 4).

**Table 4.** The association between sarcopenia and clinicopathological features of NPC.

Clinicopathological features	No. of studies	No. of patients	Effects model	OR (95%CI)	*p*	Heterogeneity	
						*I*^2^ (%)	Ph
Gender (male vs. female)	7	4652	Random	0.87 (0.42–1.77)	.694	95.5	<0.001
T stage (T3–T4 vs. T1–T2)	6	3345	Fixed	1.33 (1.12–1.59)	.002	32.0	0.195
N stage (N2–N3 vs. N0–N1)	6	3345	Fixed	1.00 (0.87–1.16)	.952	25.9	0.240
TNM stage (III–IV vs. I–II)	4	3532	Fixed	1.17 (0.94–1.46)	.153	0	0.734

TNM: tumor-node-metastasis; NPC: nasopharyngeal carcinoma.

### Sensitivity analysis

**Figure 5.**
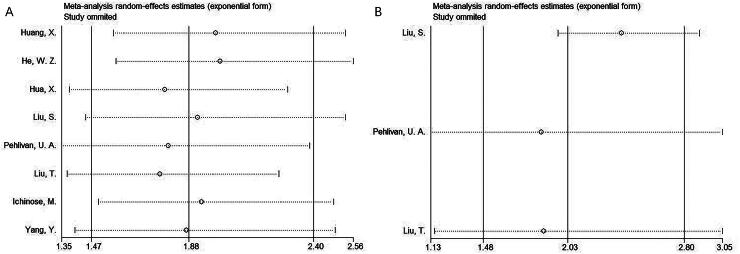
Sensitivity analysis. (A) OS and (B) PFS. According to our findings, our meta-analysis outcomes remained consistent and were not notably swayed by any one study.

Studies were removed sequentially in sensitivity analysis to analyse how every article affected overall outcome. According to our findings, our meta-analysis outcomes remained consistent and were not notably swayed by any one study (Figure 5).

### Publication bias

**Figure 6.**
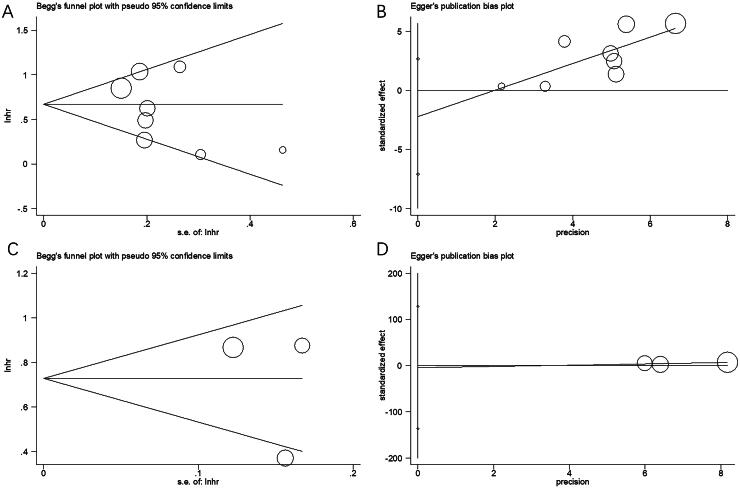
Publication bias. (A) Begg’s test for OS, *p* = .536; (B) Egger’s test for OS, *p* = .307; (C) Begg’s test for PFS, *p* = 1.000; and (D) Egger’s test for PFS, *p* = .737.

Begg’s and Egger’s tests were adopted for assessing possible publication bias, which revealed no distinct publication bias related to OS (*p* = .536/.307 by Begg’s/Egger’s tests) or PFS (*p* = 1.000/.737 by Begg’s/Egger’s tests) (Figure 6).

## Discussion

Sarcopenia has a conflicting effect on predicting NPC prognosis. This meta-analysis aggregated data from eight studies with 5458 patients for analysing its accurate effect on predicting NPC prognosis. Our results indicated sarcopenia as the distinct marker of the poor OS and PFS of NPC. Moreover, sarcopenia was significantly associated with higher T stage of NPC. Subgroup and sensitivity analyses verified that the findings were stable. Taken together, this meta-analysis showed that presence of sarcopenia was significantly related to poor short- and long-time survival in NPC. To our knowledge, the current work provides the first meta-analysis investigating the value of sarcopenia for predicting NPC prognosis.

Various factors contribute to mortality in patients with tumours and sarcopenia who have undergone radio- and/or chemotherapy. The mechanisms related to sarcopenia’s effect on predicting NPC prognosis remain to be further explored, yet they are interpreted as follows. First, sarcopenia, a disease associated with aging, leads to physiological alterations like shifts in body composition, metabolic ability and receptor activity, significantly affecting drug pharmacokinetics and pharmacodynamics [25]. Alterations in body composition due to sarcopenia might influence drug metabolism and distribution, which can affect how effective and toxic drugs are. Second, cancer patients’ muscle mass loss is related to their immune status. Muscle deterioration can affect the immune system’s ability to function. The immune system is important for fighting against cancer cells while avoiding infections [26]. If the immune system weakens, patients become vulnerable to infections and other complications, influencing their prognosis [27]. Third, sarcopenia patients could potentially have less total blood volume than individuals who are not affected by the condition. Therefore, individuals with sarcopenia could undergo more significant blood loss, leading to the need for blood transfusions during surgical procedures [28]. Therefore, sarcopenia can be the reliable marker for predicting NPC prognosis, based on the biological changes caused by sarcopenia.

Notably, the heterogeneity for PFS analysis was significant (*I*^2^ = 72.6%, *p* = .026). The heterogeneity was large and can be explained in the following aspects. First, all included studies were of retrospective design. Therefore, the inherent heterogeneity could exist due to the nature retrospective studies. Second, the diagnostic methods for sarcopenia are not uniform in included studies, which can be a source of heterogeneity (Table 1). For example, some studies used SMI and others adopted TMMV (Table 1). Third, the cut-off values for sarcopenia definition are various in eligible studies (Table 1).

Importantly, sarcopenia could predict prognosis in NPC, as shown in this meta-analysis. For patients with head and neck cancers (HNCs) undergoing radiotherapy (RT), one of the most serious complications is mandibular osteoradionecrosis (ORN) [29]. Recent research has produced a nomogram that enables individualized risk evaluation, assisting in the optimization of treatment for HNC [29]. These results highlight the importance of meticulous RT planning and proactive dental care to reduce ORN risk and enhance the quality of life for patients undergoing RT for HNC [29].

This meta-analysis reported the significant prognostic role of sarcopenia in NPC. In clinical settings, RT is the fundamental treatment approach for NPC [30]. Importantly, screening for sarcopenia can guide the planning of RT, nutritional support or early rehabilitation efforts. First, our findings propose that nutritional assessments become a part of regular cancer care to tailor treatment strategies and improve patient outcomes. Second, according to a recent study, smaller psoas size was significantly correlated with reduced OS and PFS in NSCLC patients undergoing radiation treatment [31]. Therefore, nutrition support should be emphasized during RT treatment. Third, for patients with cancer, sarcopenia could also serve as an indicator for tumour recurrence during follow-up [32].

Recently, sarcopenia is widely suggested with a prominent effect on predicting tumour survival in meta-analysis [33–37]. Wang et al. reported from the meta-analysis involving 1907 cases that pretreatment sarcopenia and muscle change during treatment are closely related to both OS and disease progression of cervical cancer [33]. As reported by Keshavjee et al., preoperative sarcopenia was related to poor postoperative outcomes in colorectal cancer patients in a meta-analysis including 40 studies [34]. A recent meta-analysis indicated that sarcopenia was negatively correlated with survival outcomes in patients with endometrial cancer [35]. Zeng et al. conducted the meta-analysis with 26 studies and revealed the significant link of sarcopenia to poor OS, recurrence-free survival and cancer-specific survival of bladder cancer [36]. As mentioned by Liu et al., preoperative sarcopenia closely related to inferior prognosis in pancreatic cancer after surgery through a meta-analysis consisting of 5888 patients [37]. The results of the current meta-analysis are in line with findings on other cancer types, which suggest that sarcopenia can be a significant prognostic marker in various cancers.

There were certain limitations in the present study. First, the recruited studies were retrospective. Consequently, there is still selection bias considering the retrospective nature. Second, the thresholds for identifying sarcopenia are non-uniform across included studies. Third, most eligible studies were conducted in Asia, especially China (six of eight). There is a risk of selection bias due to the large number of studies from Asia and the limited diversity in geographic representation. Although we comprehensively searched the literature and only included English publications, this may limit generalizability of our results. And we highlight the importance of conducting validation in non-endemic regions. Fourth, publication bias may still exist, because only publications in English were included. Moreover, only studies could be publicly searched and available are retrieved in this meta-analysis. Given the above limitations, more international collaborations on multi-regional prospective studies with uniform SMI threshold should be conducted for validation.

## Conclusions

This meta-analysis demonstrated that sarcopenia served as the marker to predict unfavourable OS and PFS of NPC cases. Moreover, sarcopenia is also significantly associated with higher T stage of NPC.

## Supplementary Material

PRISMA_2020_checklist.docx

Supplemental file 1.docx

## Data Availability

The data that support the findings of this study are available from the corresponding author upon reasonable request.
