# Supplementary material for: Prognostic value of sarcopenia in patients with nasopharyngeal carcinoma: a meta-analysis
Source: Ann Med. 2025 Jul 11;57(1):2530695. doi: 10.1080/07853890.2025.2530695 (PMC12258165; doi:10.1080/07853890.2025.2530695)
Supplement: Supplemental file 1.docx [file IANN_A_2530695_SM2393.docx]

Supplemental file 1 The quality assessment scores by NOS of included studies in this meta-analysis.

| Study | Year | Selection (0-4 points) | | | | Comparability  (0-2 points) | Outcome  (0-3 points) | | | Total score |
| --- | --- | --- | --- | --- | --- | --- | --- | --- | --- | --- |
|  |  | Representativeness of the exposed cohort | Selection of the non exposed cohort | Ascertainment of exposure | Demonstration that outcome of interest was not present at start of study | Comparability of cohorts on the basis of the design or analysis | Assessment of outcome | Was follow-up long enough for outcomes to occur | Adequacy of follow up of cohorts |  |
| Huang, X. | 2019 | ★ | ★ | ★ | ★ | ★☆ | ★ | ★ | ★ | 8 |
| He, W. Z. | 2020 | ★ | ★ | ★ | ★ | ★★ | ★ | ★ | ★ | 9 |
| Hua, X. | 2021 | ★ | ★ | ★ | ★ | ★☆ | ★ | ★ | ★ | 8 |
| Liu, S. | 2023 | ★ | ★ | ★ | ★ | ★★ | ★ | ★ | ★ | 9 |
| Pehlivan, U. A. | 2023 | ★ | ★ | ☆ | ★ | ★☆ | ★ | ★ | ★ | 7 |
| Liu, T. | 2024 | ★ | ★ | ★ | ★ | ★★ | ★ | ☆ | ★ | 8 |
| Ichinose, M. | 2025 | ★ | ★ | ☆ | ★ | ★★ | ★ | ★ | ★ | 8 |
| Yang, Y. | 2025 | ★ | ★ | ★ | ★ | ★☆ | ★ | ★ | ★ | 8 |

NOS: Newcastle-Ottawa Scale; a ★ represents 1 point; a ☆ represents 0 point.
